# Supplementary material for: Loss-of-function mutations in the dystonia gene THAP1 impair proteasome function by inhibiting PSMB5 expression
Source: Nat Commun. 2025 Feb 10;16:1511. doi: 10.1038/s41467-025-56782-1 (PMC11811203; doi:10.1038/s41467-025-56782-1)
Supplement: Supplementary file 1 — Supplementary Information [file 41467_2025_56782_MOESM1_ESM.pdf]

SUPPLEMENTARY INFORMATION

Loss-of-function mutations in the dystonia gene THAP1 impair proteasome function by inhibiting PSMB5 expression

Dylan E. Ramage[1], Drew W. Grant[1], Richard T. Timms[1*]

[1]Cambridge Institute of Therapeutic Immunology and Infectious Disease, Department of Medicine, University of Cambridge, Puddicombe Way, Cambridge, CB2 0AW, UK

*Correspondence: rtt20@cam.ac.uk

a

Rank	Gene1	Gene2	GLS_F	GLS_R	P-value	Direction	Annotation
#1	UBA5	UFM1	0.9157	0.6459	4.00E-95	+	UFMylation
#2	PSMG1	PSMG2	0.6580	0.8519	1.00E-87	+	Proteasome
#3	UFM1	UFL1	0.5439	0.9653	2.00E-79	+	UFMylation
#4	DET1	RFWD2	0.7773	0.6574	2.00E-76	+	COP1 E3 ligase complex
#5	FBXO42	CCDC6	0.5732	0.8861	8.00E-76	+	Cul1 complex
#6	UBA5	UFL1	0.6229	0.7796	4.00E-71	+	UFMylation
#7	UBR4	KCMF1	0.6678	0.7239	1.00E-70	+	SIFI E3 ligase complex
#8	WDR26	YPEL5	0.9263	0.5127	6.00E-69	+	CTLH E3 ligase complex
#9	UBE2H	YPEL5	0.8087	0.5596	1.00E-64	+	CTLH E3 ligase complex
#10	MAEA	UBE2H	0.7447	0.6044	3.00E-64	+	CTLH E3 ligase complex
#11	ARIH2	CUL5	0.5902	0.7623	4.00E-64	+	Cul5 complex
#12	DDI2	NGLY1	0.5733	0.7698	2.00E-62	+	Nrf1 pathway
#13	UFSP2	UBA5	0.7353	0.5992	2.00E-62	+	UFMylation
#14	WDR24	MIOS	0.6474	0.6778	4.00E-62	+	GATOR2 complex
#15	URM1	CTU2	0.7035	0.6156	6.00E-61	+	UFMylation
#16	FANCL	FANCG	0.7221	0.5848	5.00E-59	+	Fanconi anemia pathway
#17	UFM1	UFSP2	0.4916	0.8554	8.00E-59	+	UFMylation
#18	ARIH2	UBE2F	0.6943	0.5985	8.00E-58	+	Cul5 complex
#19	FANCL	FANCI	0.7055	0.5889	9.00E-58	+	Fanconi anemia ID2 complex
#20	ARNT	AHR	0.6797	0.6107	9.00E-58	+	Aryl hydrocarbon receptor complex
#21	UBE2H	WDR26	0.5684	0.7106	1.00E-55	+	CTLH E3 ligase complex
#22	UBE2F	CUL5	0.5186	0.7770	1.00E-55	+	Cul5 complex
#23	PEX12	PEX6	0.7792	0.5152	2.00E-55	+	Peroxisome biogenesis
#24	UFL1	UFSP2	0.6358	0.6233	2.00E-54	+	UFMylation
#25	UBA5	C1orf27	0.4456	0.8620	3.00E-52	+	UFMylation
#26	UBE2T	FANCI	0.6883	0.5468	5.00E-51	+	Fanconi anemia pathway
#27	WDR26	MAEA	0.6147	0.6058	2.00E-50	+	CTLH E3 ligase complex
#28	UFSP2	C1orf27	0.4852	0.7649	3.00E-50	+	UFMylation
#29	PEX2	PEX6	0.7560	0.4835	3.00E-49	+	Peroxisome biogenesis
#30	SKP2	CKS1B	0.4598	0.7892	8.00E-49	+	Cul1 complex
#31	VPS41	VPS39	0.5880	0.6061	7.00E-48	+	HOPS complex
#32	WDR48	USP1	0.4315	0.8176	3.00E-47	+	DUB complex
#33	WDR59	MIOS	0.8302	0.4191	2.00E-46	+	GATOR2 complex
#34	UFC1	UBA5	0.8057	0.4311	3.00E-46	+	UFMylation
#35	FANCL	FANCF	0.7247	0.4796	3.00E-46	+	Fanconi anemia pathway
#36	MAEA	YPEL5	0.7820	0.4392	1.00E-45	+	CTLH E3 ligase complex
#37	DDI2	NFE2L1	0.4513	0.7419	2.00E-44	+	Nrf1 pathway
#38	UFL1	C1orf27	0.4610	0.7124	3.00E-43	+	UFMylation
#39	PEX2	PEX5	0.5828	0.5590	6.00E-43	+	Peroxisome biogenesis
#40	PEX10	PEX6	0.7032	0.4621	7.00E-43	+	Peroxisome biogenesis
#41	BIRC6	UBA6	0.4346	0.7460	1.00E-42	+	Negative regulation of autophagy
#42	UFC1	UFM1	0.9245	0.3489	2.00E-42	+	UFMylation
#43	RNF31	MAP3K7	0.7035	0.4528	9.00E-42	+	LUBAC complex
#44	PSMB5	THAP1	0.5436	0.5845	1.00E-41	+	This paper

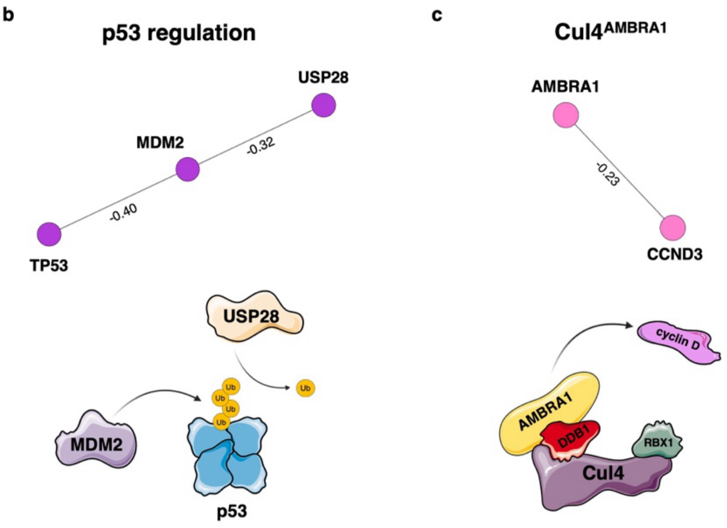

Supplementary Figure 1 | Co-essentiality analysis defines functional relationships amongst UPS genes.

(A) Co-essential relationships involving UPS genes reflect functional relationships. The table illustrates the strongest pairwise relationships involving at least one gene known to function as part of the UPS, based on previous analysis of DepMap data[16]. The co-essential relationship between PSMB5 and THAP1 (highlighted in green) represents the most significant unexplained relationship across this dataset.

(B-C) Negative co-essential relationships highlight known E3 ligase-substrate pairs, including p53 which is targeted by MDM2[34] (B) and cyclin D which is degraded by Cul4^{AMBRA1}[35–37] (C). Source data are provided as a Source Data File.

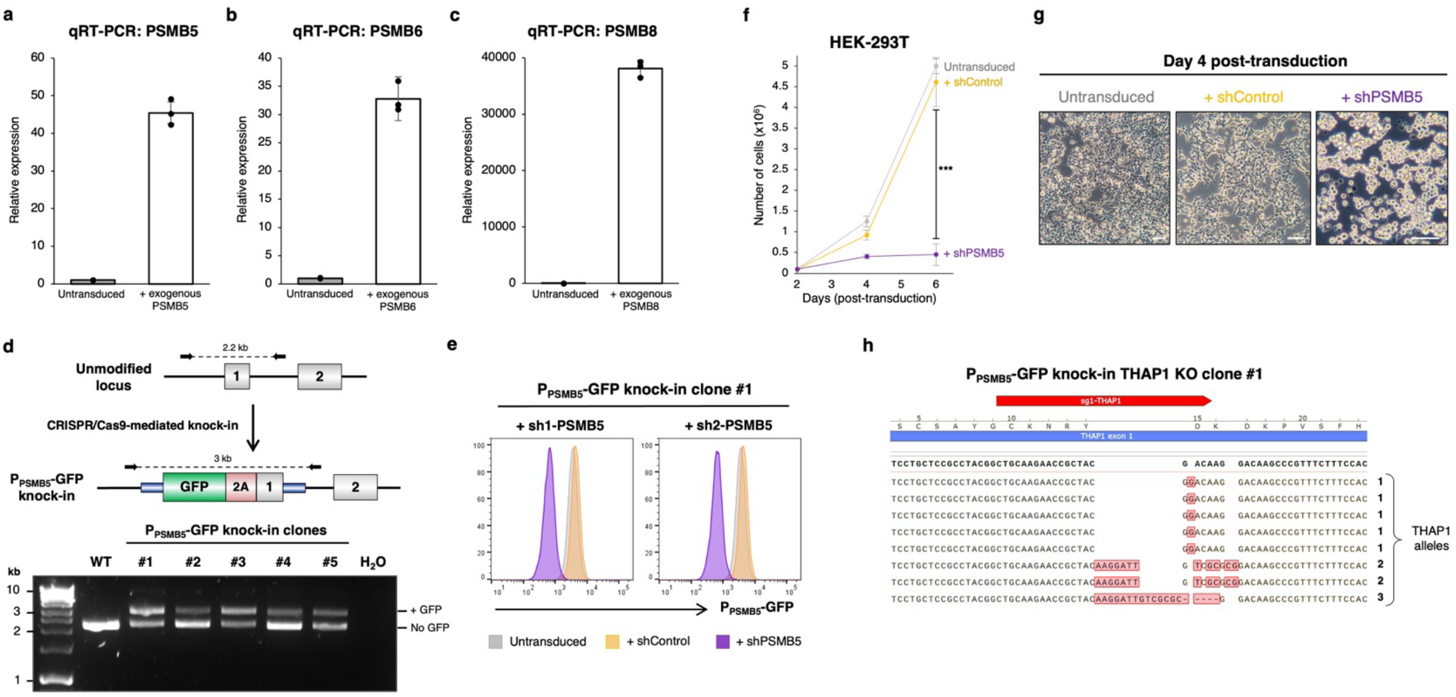

Supplementary Figure 2 | Validation of manipulated HEK-293T cell lines.

(A-C) Validation of exogenous expression of proteasome subunits. Lentiviral expression of PSMB5 (A), PSMB6 (B) and PSMB8 (C) was validated by qRT-PCR. Data are presented as mean values of n=3 technical replicates +/- s.d.

(D) Validation of P_{PSMB5}-GFP reporter clones by PCR from genomic DNA.

(E) P_{PSMB5}-GFP reporter cells faithfully monitor PSMB5 expression. A P_{PSMB5}-GFP knock-in clone was transduced with two shRNAs targeting PSMB5 and P_{PSMB5}-GFP expression was measured by flow cytometry.

(F-G) PSMB5 knockdown is toxic in HEK-293T cells. Cells were transduced with a lentiviral vector expressing the indicated shRNAs. 48 hours post-transduction, cells were counted, plated in equal numbers, and their viability assessed by counting (F) and brightfield microscopy (G). Data in (F) are presented as mean values of n=3 biological replicates +/- s.d. (***$P < 0.001$, two-tailed t-test) (Scale bar = 100 µm)

(H) Genomic characterization of THAP1 KO clone #1. PCR amplification of the sgRNA target site from genomic DNA followed by TOPO cloning and Sanger sequencing revealed three mutated THAP1 alleles.

Source data are provided as a Source Data File.

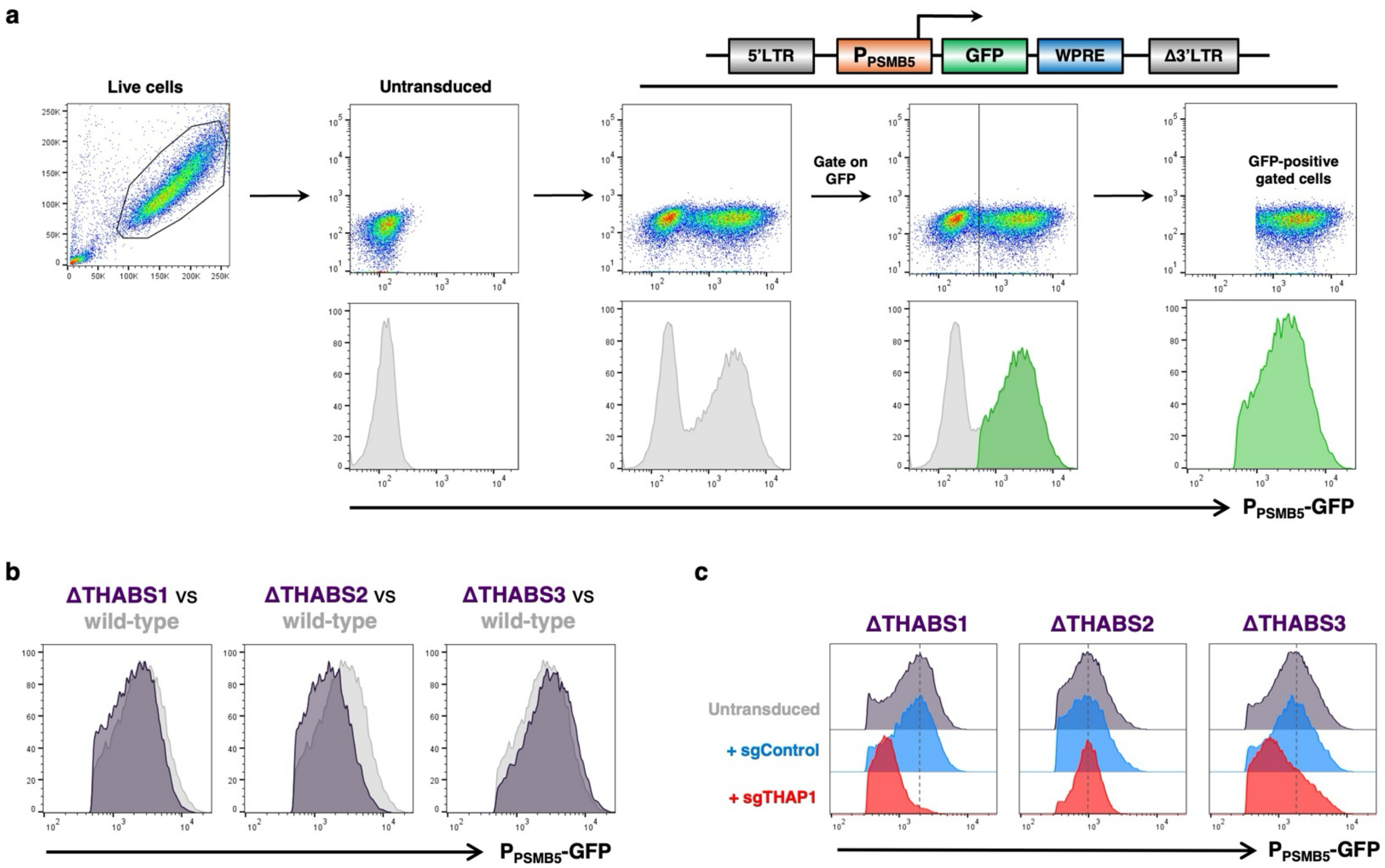

Supplementary Figure 3 | THAP1 acts through a cognate site in the PSMB5 promoter.

(A) Schematic depiction of the gating strategy used to assess GFP expression from the reporter vector. Following transduction with the P_{PSMB5}-GFP lentiviral reporter vector at single copy, GFP^{+} cells were gated for downstream analysis.

(B-C) THAP1 activates the PSMB5 promoter through binding to THABS site 2. Deletion of THABS2, but not THABS1 or THABS3, reduced expression from P_{PSMB5}-GFP lentiviral reporter (B); furthermore, THAP1 disruption decreased expression from the ΔTHABS1 and ΔTHABS3 vectors, but did not further decrease expression from the ΔTHABS2 vector (C).

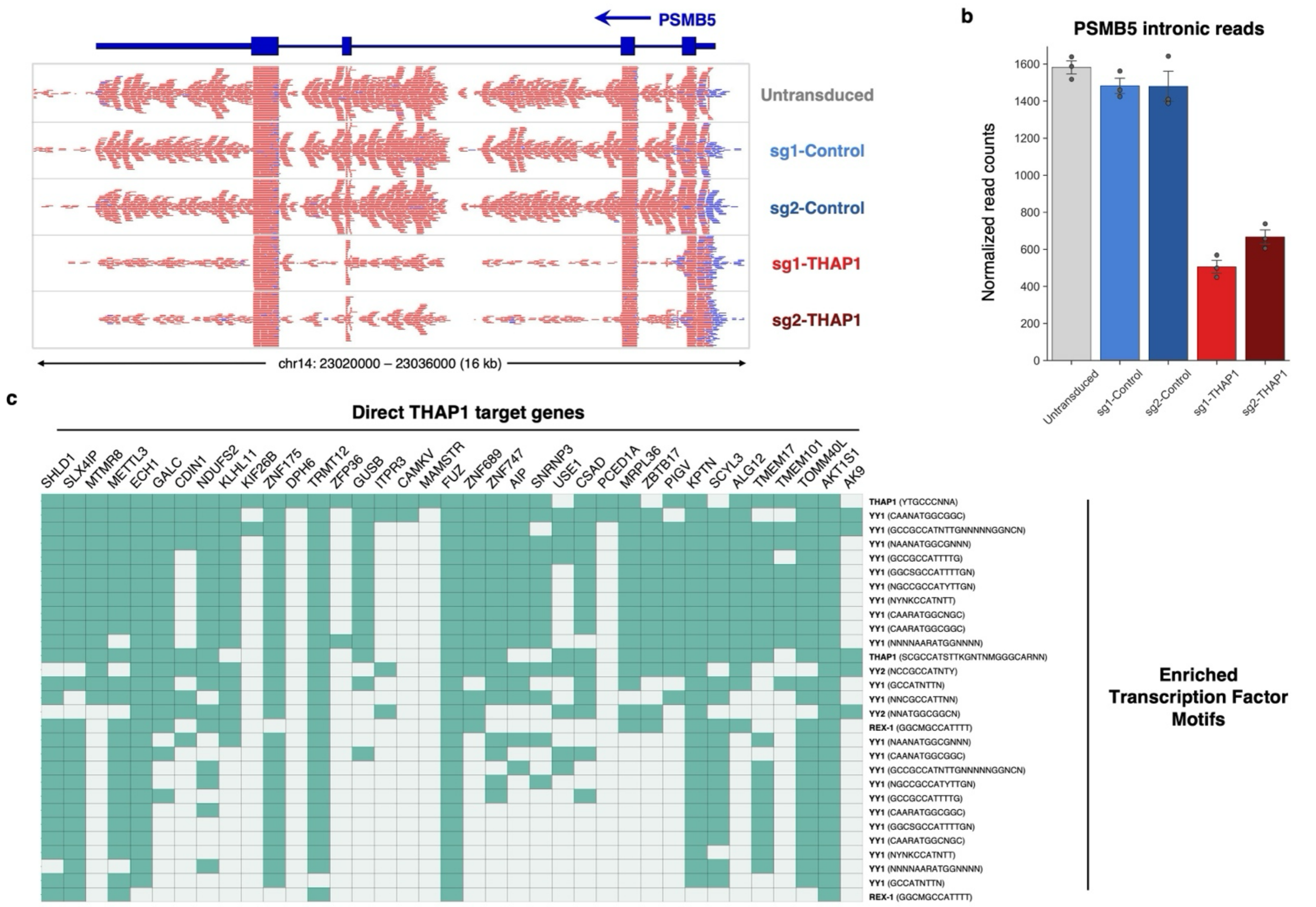

Supplementary Figure 4 | Analysis of THAP1 target genes identified by RNA-seq analysis.

(A-B) Transcriptional downregulation of PSMB5 upon loss of THAP1. Owing to the necessity to overexpress PSMB5 to maintain the viability of THAP1 knockout cells, PSMB5 was not identified as a differentially expressed gene by RNA-seq analysis; however, reads mapping to intronic sequences (which are not present in the exogenous PSMB5 expression construct) show that endogenous PSMB5 expression was markedly reduced in THAP1 knockout cells. Raw sequence reads are shown in (A) and quantified in (B).

(C) Transcription factor binding sites enriched in the promoters of THAP1 target genes. Analysis using g:Profiler[71] identified significant enrichment for the indicated motifs; the presence of the motif in the promoter of the direct THAP1 targets genes is indicated by the green cells.

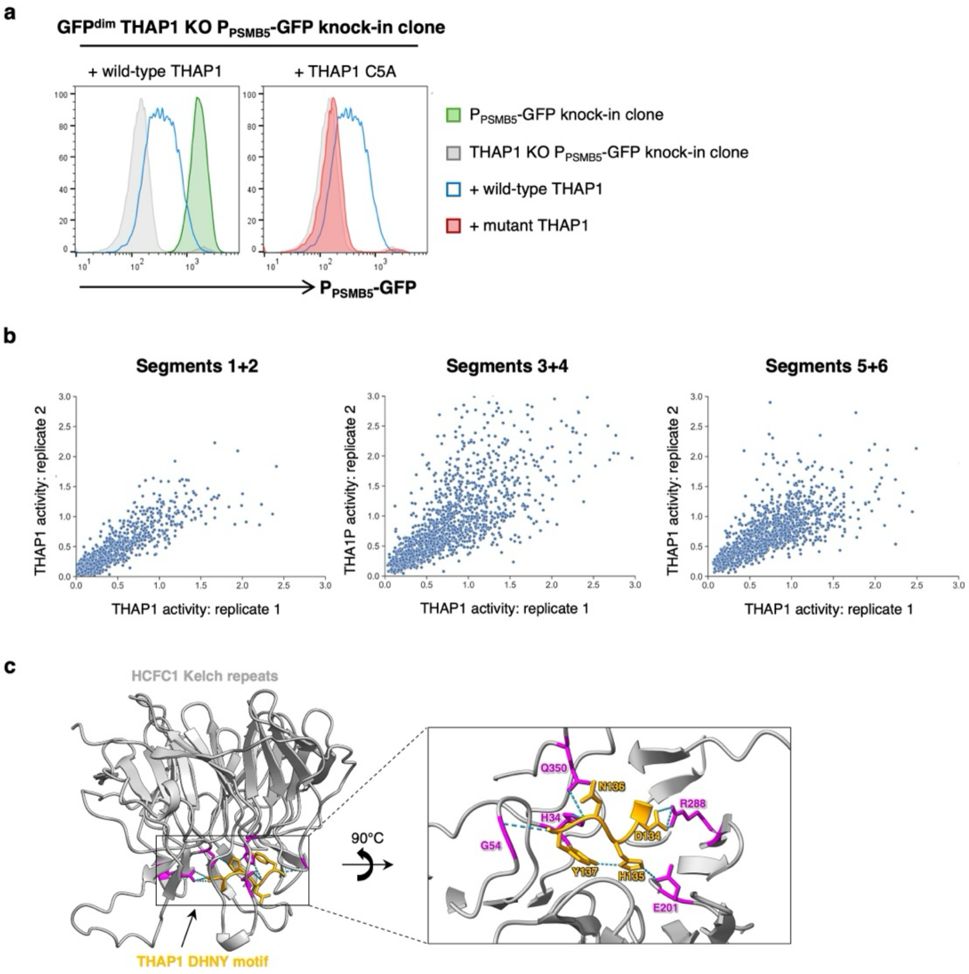

Supplementary Figure 5 | A deep mutagenic scan defines the functional consequences of THAP1 mutations.

(A) Genetic complementation of THAP1 knockout cells. A THAP1 knockout P_{PSMB5}-GFP reporter clone was transduced with a lentiviral vector expressing either wild-type or mutant (C5A, which disrupts zinc coordination by the THAP-type zinc finger) THAP1, and restoration of PSMB5 reporter expression assayed by flow cytometry.

(B) Assessing concordance between replicate experiments. The THAP1 scan was performed in duplicate: scatterplots compare the activity of each individual THAP1 mutant construct (blue dots) between the two replicates.

(C) AlphaFold 3 prediction of the interaction between the THAP1 DHNY motif (gold) and residues (magenta) of the HCFC1 kelch repeats. Hydrogen bonds (cyan) predicted by ChimeraX are shown.

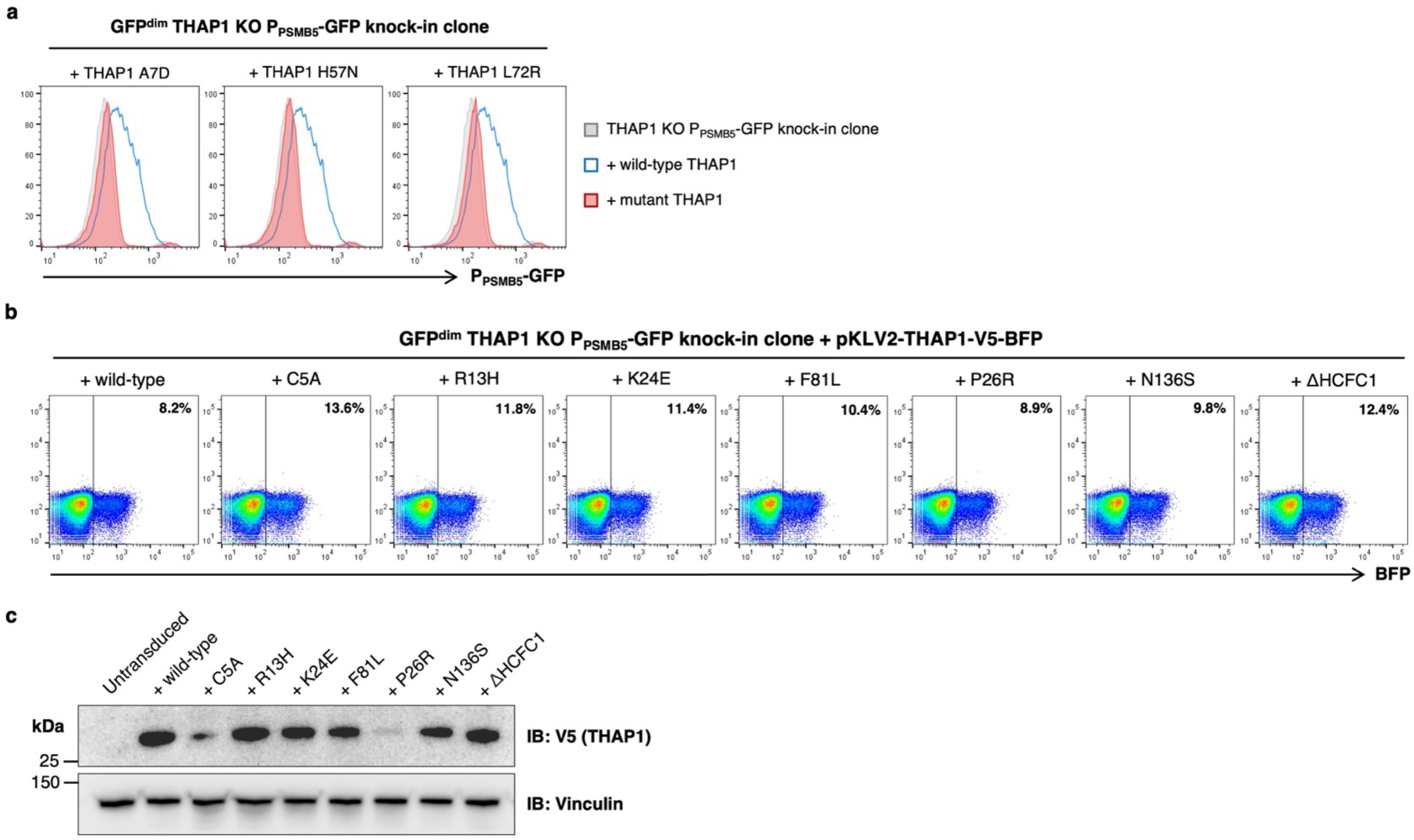

Supplementary Figure 6 | Assessing the functional impact of THAP1 mutations found in Dystonia patients.

(A) Validation of the scan results. Individual validation by flow cytometry for three additional THAP1 mutants is shown, all of which the screen suggests should be inactive.

(B-C) Validation of the expression of a panel of inactive mutants. For seven inactive mutants, we generated constructs appending a C-terminal V5 epitope tag to allow detection by immunoblot. Following single-copy transduction of HEK-293T cells (~10% BFP^{+}) in each case (**B**), protein abundance was assessed by immunoblot **(C)**. With the exception of the C5A and P26R mutants, all were expressed at approximately the same level as the wild-type protein. Immunoblot data is representative of at least two independent experiments.

Source data are provided as a Source Data File.

SUPPLEMENTARY TABLE LEGENDS

Supplementary Table 1 | THAP1 ChIP-seq peaks. THAP1 ChIP-seq data from the ENCODE project was obtained from GSM803408. Peaks were identified using the implementation of the MACS peak caller in SeqMonk, using a P-value cutoff of 10^{-16} and a fragment size of 150 bp.

Supplementary Table 2 | Effect of THAP1 loss on the transcriptome as assessed by RNA-seq.

Supplementary Table 3 | A deep mutational scan of THAP1.

Supplementary Table 4 | Primer sequences.

SUPPLEMENTARY INFORMATION REFERENCES

71. Raudvere, U. et al. g:Profiler: a web server for functional enrichment analysis and conversions of gene lists (2019 update). Nucleic Acids Res 47, W191–W198 (2019).
