# Supplementary material for: Loss-of-function mutations in the dystonia gene THAP1 impair proteasome function by inhibiting PSMB5 expression
Source: Nat Commun. 2025 Feb 10;16:1511. doi: 10.1038/s41467-025-56782-1 (PMC11811203; doi:10.1038/s41467-025-56782-1)
Supplement: Supplementary file 6 — Reporting Summary [file 41467_2025_56782_MOESM6_ESM.pdf]

nature portfolio

Corresponding author(s): Timms, RT

Last updated by author(s): Jan 17, 2025

Reporting Summary

Nature Portfolio wishes to improve the reproducibility of the work that we publish. This form provides structure for consistency and transparency in reporting. For further information on Nature Portfolio policies, see our Editorial Policies and the Editorial Policy Checklist.

Statistics

For all statistical analyses, confirm that the following items are present in the figure legend, table legend, main text, or Methods section.

n/a	Confirmed	
☐	☒	The exact sample size (*n*) for each experimental group/condition, given as a discrete number and unit of measurement
☐	☒	A statement on whether measurements were taken from distinct samples or whether the same sample was measured repeatedly
☐	☒	The statistical test(s) used AND whether they are one- or two-sided<br>*Only common tests should be described solely by name; describe more complex techniques in the Methods section.*
☒	☐	A description of all covariates tested
☒	☐	A description of any assumptions or corrections, such as tests of normality and adjustment for multiple comparisons
☐	☒	A full description of the statistical parameters including central tendency (e.g. means) or other basic estimates (e.g. regression coefficient) AND variation (e.g. standard deviation) or associated estimates of uncertainty (e.g. confidence intervals)
☐	☒	For null hypothesis testing, the test statistic (e.g. F, t, r) with confidence intervals, effect sizes, degrees of freedom and P value noted<br>*Give P values as exact values whenever suitable.*
☒	☐	For Bayesian analysis, information on the choice of priors and Markov chain Monte Carlo settings
☒	☐	For hierarchical and complex designs, identification of the appropriate level for tests and full reporting of outcomes
☒	☐	Estimates of effect sizes (e.g. Cohen's *d*, Pearson's *r*), indicating how they were calculated

Our web collection on statistics for biologists contains articles on many of the points above.

Software and code

Policy information about availability of computer code

Data collection	Flow cytometry data was collected using BD FACSDiva (8.0.3).
Data analysis	Analysis of Illumina sequencing data was carried out using Cutadapt (v4.1), HISAT2 (2.2.1) and Bowtie 2 (2.4.1). RNA-seq data was further analyzed and visualized in SeqMonk (1.48.1). ChIP-seq data was visualized in IGV (2.16.0). Data analysis and visualization was performed in Python (3.6) using the Pandas (1.5.1), Matplotlib (3.6.0) and Seaborn (v0.12) libraries. Analysis of immunoblots was performed using GIMP (2.10.34). Structural analysis was performed using ChimeraX-1.6. Flow cytometry data was analyzed using FlowJo (10.4).

For manuscripts utilizing custom algorithms or software that are central to the research but not yet described in published literature, software must be made available to editors and reviewers. We strongly encourage code deposition in a community repository (e.g. GitHub). See the Nature Portfolio guidelines for submitting code & software for further information.

Data

Policy information about availability of data

All manuscripts must include a data availability statement. This statement should provide the following information, where applicable:

- Accession codes, unique identifiers, or web links for publicly available datasets
- A description of any restrictions on data availability
- For clinical datasets or third party data, please ensure that the statement adheres to our policy

THAP1 RNA-seq data has been deposited at GEO (GSE264536; https://www.ncbi.nlm.nih.gov/geo/query/acc.cgi?acc=GSE264536) and Illumina sequencing data from the THAP1 deep mutagenic scan are available at SRA (PRJNA1102672; https://www.ncbi.nlm.nih.gov/sra/PRJNA1102672). THAP1 ChIP-seq data was obtained

from GEO (GSM803408; https://www.ncbi.nlm.nih.gov/geo/query/acc.cgi?acc=GSM803408). DepMap datasets are publicly available at https://depmap.org/portal/download/all/. Source data are provided with this paper as a Source Data file.

Research involving human participants, their data, or biological material

Policy information about studies with human participants or human data. See also policy information about sex, gender (identity/presentation), and sexual orientation and race, ethnicity and racism.

Reporting on sex and gender	N/A
Reporting on race, ethnicity, or other socially relevant groupings	N/A
Population characteristics	N/A
Recruitment	N/A
Ethics oversight	N/A

Note that full information on the approval of the study protocol must also be provided in the manuscript.

Field-specific reporting

Please select the one below that is the best fit for your research. If you are not sure, read the appropriate sections before making your selection.

☒ Life sciences ☐ Behavioural & social sciences ☐ Ecological, evolutionary & environmental sciences

For a reference copy of the document with all sections, see nature.com/documents/nr-reporting-summary-flat.pdf

Life sciences study design

All studies must disclose on these points even when the disclosure is negative.

Sample size	No sample size calculations were performed. Saturation mutagenesis screening experiments were performed at sufficient scale to maintain at least 100-fold representation of the library and a minimum of 10,000 live cells were analyzed for all flow cytometry experiments, as these are used as standard in the field.
Data exclusions	No data were excluded.
Replication	Experiments monitoring cell viability were performed in biological triplicate. All qRT-PCR experiments were performed in technical triplicate with the data shown being representative of at least two individual experiments. All flow cytometry data is representative of at least two independent experiments. The RNA-seq experiment was performed in biological triplicate, using two different sgRNAs targeting THAP1. The deep mutagenic scan of THAP1 was performed in biological duplicate and the critical findings were validated through independent experiments involving single vector lentiviral trasnductions. Attempts at replication were successful.
Randomization	The study design did not permit randomization to be employed.
Blinding	The study design did not permit blinding to be employed.

Reporting for specific materials, systems and methods

We require information from authors about some types of materials, experimental systems and methods used in many studies. Here, indicate whether each material, system or method listed is relevant to your study. If you are not sure if a list item applies to your research, read the appropriate section before selecting a response.

Materials & experimental systems

n/a	Involved in the study	
☐	☒	Antibodies
☐	☒	Eukaryotic cell lines
☒	☐	Palaeontology and archaeology
☒	☐	Animals and other organisms
☒	☐	Clinical data
☒	☐	Dual use research of concern
☒	☐	Plants

Methods

n/a	Involved in the study	
☒	☐	ChIP-seq
☐	☒	Flow cytometry
☒	☐	MRI-based neuroimaging

Antibodies

Antibodies used	Rabbit anti-THAP1 (Proteintech, #12584-1-AP), diluted 1:5000<br>Mouse anti-V5 tag (Abcam, #AB27671), diluted 1:10000<br>Rabbit anti-PSMB5 (Enzo Life Sciences, #BML-PW8895), diluted 1:5000<br>Mouse anti-PSMB6 (Enzo Life Sciences, #BML-PW8140), diluted 1:5000<br>Mouse anti-PSMB7 (Enzo Life Sciences, #BML-PW8145), diluted 1:5000<br>Mouse anti-HIF-1α (BD, #610959), diluted 1:1000<br>Mouse anti-Proteasome 20S alpha 1+2+3+5+6+7 antibody (Abcam, #22674), diluted 1:1000<br>Mouse anti-Vinculin (Sigma, #V9131), diluted 1:10000<br>Mouse anti-β-actin (Sigma, #A2228), diluted 1:10000
Validation	With the exception of the anti-THAP1 antibody whose specificity we assessed in Fig. 3G, validation data for all of the above antibodies can be found at the following manufacturer websites:<br>https://www.abcam.com/en-us/products/primary-antibodies/v5-tag-antibody-sv5-pk1-ab27671<br>https://www.enzo.com/product/proteasome-20s-%CE%B25-subunit-human-polyclonal-antibody/<br>https://www.enzo.com/product/proteasome-20s-%CE%B21-subunit-monoclonal-antibody-mcp421/<br>https://www.enzo.com/product/proteasome-20s-%CE%B22-subunit-monoclonal-antibody-mcp168/<br>https://www.bdbiosciences.com/en-gb/products/reagents/microscopy-imaging-reagents/immunofluorescence-reagents/purified-mouse-anti-human-hif-1.610959<br>https://www.abcam.com/en-us/products/primary-antibodies/proteasome-20s-alpha-123567-antibody-mcp231-ab22674<br>https://www.sigmaaldrich.com/GB/en/product/sigma/v9131<br>srsltid=AfmBOoonRoFCDvLM6FHNB4g7a2lPVU85jT_PBn_dL8MRixyxL4QhLK3a<br>https://www.sigmaaldrich.com/GB/en/product/sigma/a2228?<br>srsltid=AfmBOoprmorQL2NUBu5sFqMrXlGJPcny1n_0EQqDhdCWIao0p2--HUSc

Eukaryotic cell lines

Policy information about cell lines and Sex and Gender in Research

Cell line source(s)	HEK293T cells were obtained from ATCC.
Authentication	Authenticated HEK293T cells were obtained from ATCC; we did not perform any additional authentication.
Mycoplasma contamination	Cells were routinely tested for Mycoplasma contamination and found to be negative.
Commonly misidentified lines<br>(See ICLAC register)	No commonly misidentified cell lines were used.

Plants

Seed stocks	N/A
Novel plant genotypes	N/A
Authentication	N/A

Flow Cytometry

Plots

Confirm that:

- [x] The axis labels state the marker and fluorochrome used (e.g. CD4-FITC).
- [x] The axis scales are clearly visible. Include numbers along axes only for bottom left plot of group (a 'group' is an analysis of identical markers).
- [ ] All plots are contour plots with outliers or pseudocolor plots.
- [x] A numerical value for number of cells or percentage (with statistics) is provided.

Methodology

Sample preparation	HEK293T cells were trypsinized, washed once with PBS, and aliquoted into 5 ml FACS tubes.
Instrument	BD LSR II
Software	Data was collected using FACS DIVA and analyzed using FlowJo.
Cell population abundance	Live HEK293T cells were gated based on forward and side scatter and typically represented >80% of all events.
Gating strategy	PSMB5-GFP reporter knock-in cells were isolated by FACS based on GFP positivity. Gating for subsequent experiments assessed changes in GFP levels from a relevant parental cell population, which is outlined in the manuscript.<br>For GPS experiments, gating for DsRed+ cells ensured that only transduced cells were analyzed.

☒ Tick this box to confirm that a figure exemplifying the gating strategy is provided in the Supplementary Information.
