# Supplementary material for: Loss-of-function mutations in the dystonia gene THAP1 impair proteasome function by inhibiting PSMB5 expression
Source: Nat Commun. 2025 Feb 10;16:1511. doi: 10.1038/s41467-025-56782-1 (PMC11811203; doi:10.1038/s41467-025-56782-1)
Supplement: Supplementary file 7 — Description of Supplementary Data files [file 41467_2025_56782_MOESM7_ESM.docx]

**Description of additional Supplementary data files**

**File Name:** Supplementary Data 1

**Description:** THAP1 ChIP-seq peaks. THAP1 ChIP-seq data from the ENCODE project was obtained from GSM803408. Peaks were identified using the implementation of the MACS peak caller in SeqMonk, using a P-value cutoff of 10-16 and a fragment size of 150 bp.

**File Name:** Supplementary Data 2

**Description:** Effect of THAP1 loss on the transcriptome as assessed by RNA-seq.

**File Name:** Supplementary Data 3

**Description:** A deep mutational scan of THAP1.

**File Name**: Supplementary Table 4

**Description:** List of primer sequences used in the study.
